# Renal cell carcinoma sharply captured by imaging technology at an early stage in a hemodialysis patient: Usefulness of noninvasive monochrome superb microvascular imaging

**DOI:** 10.1111/hdi.12928

**Published:** 2021-03-29

**Authors:** Rie Ohsawa, Hiroyuki Kadoya, Atsushi Obata, Takahiro Obata, Atsuyuki Tokuyama, Tamaki Sasaki, Naoki Kashihara, Hideaki Kaneto

**Affiliations:** ^1^ Obata Medical Clinic Japan; ^2^ Department of Nephrology and Hypertension Kawasaki Medical School Kurashiki Japan; ^3^ Department of Diabetes, Endocrinology and Metabolism Kawasaki Medical School Kurashiki Japan

**Keywords:** hemodialysis, monochrome superb microvascular imaging, renal cell carcinoma, type 2 diabetes, ultrasonography

## Abstract

It has been drawing much attention that type 2 diabetes mellitus is closely associated with increased incidence of numerous cancers and their poor prognosis. Consequently, malignancy has been recently recognized as one of diabetic complications in addition to various conventional complications. Furthermore, it is well known that the prevalence of renal cell carcinoma (RCC) is drastically increased in hemodialysis (HD) patients. Therefore, screening of RCCs in HD patients is a very important and urgent issue as there are no highly sensitive tumor markers for RCCs. Monochrome superb microvascular imaging (mSMI) is a relatively new Doppler ultrasound technique and is useful especially when evaluating very slow blood flow state, because this allows for imaging microvessels with low velocity in the absence of a contrast agent. Thus, mSMI might be also useful when contrast enhancement is not obvious on CT and/or contrast‐enhanced ultrasonography using perflubutane or contrast agents are contraindicated. Moreover, it has been reported that mSMI could effectively detect vascularity of renal malignant tumor than benign renal mass in nondialysis patients. We propose that mSMI of ultrasonography could become one of the very useful methods for detecting RCCs at an early stage with high sensitivity in HD patients.

## INTRODUCTION

It has been drawing much attention that type 2 diabetes mellitus (T2DM) is closely associated with increased incidence of numerous cancers and their poor prognosis. It is also reported that T2DM could increase the risk of renal cell carcinoma (RCC).^1^ T2DM is also known as the leading cause of end‐stage renal disease (ESRD) and hemodialysis (HD). Furthermore, it is well known that the prevalence of RCC is drastically increased in HD patients.^2^ It is also reported that mortality of malignancy has not decreased although mortality of cardiovascular diseases is decreasing in HD patients with T2DM. In such conditions, screening of RCCs in HD patients is a very important and urgent issue as there are no highly sensitive tumor markers for RCCs. However, as renal blood perfusion is scarce in HD patients, it is often hard to detect RCCs at an early stage by conventional ultrasonographic imaging such as color or power Doppler modes. Therefore, it is often the case that RCCs are diagnosed when their size becomes relatively large in HD patients. On the other hand, monochrome superb microvascular imaging (mSMI) is a relatively new Doppler ultrasound technique and is useful especially when evaluating very slow blood flow state, because this allows for imaging microvessels with low velocity in the absence of a contrast agent.^3^ Here, we present a case of an early stage RCC, which was sharply captured by mSMI of abdominal ultrasonography (Aplio i700; Canon Medical Systems Corporation) in a HD patient with T2DM.

### Case report

**FIGURE 1:**
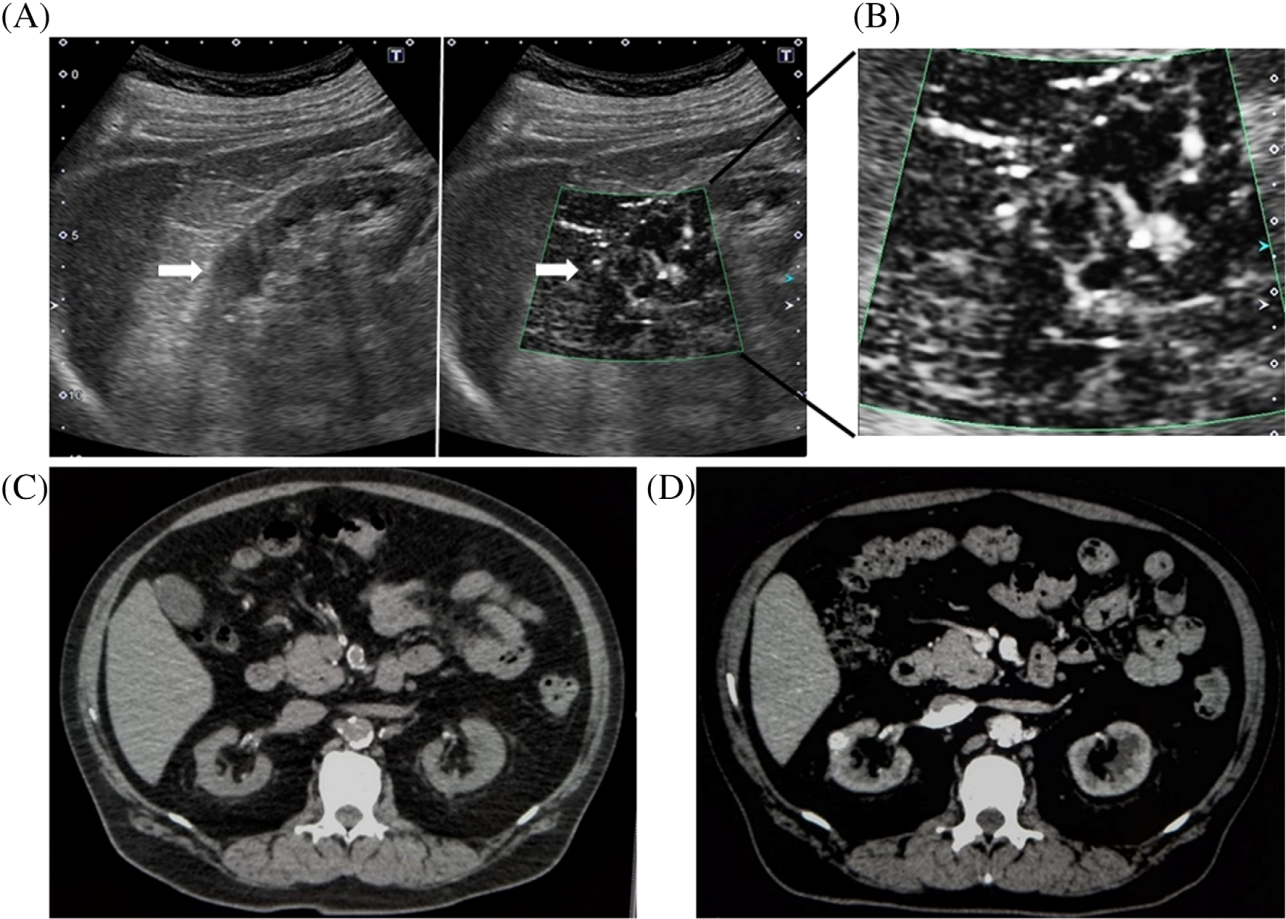
(a) B mode image in the ultrasonography. Oval mass is observed at the center part of the right kidney (17 mm × 16 mm in diameter) and it shows relatively low and homogeneous echo in the internal part (white arrow). (b) Monochrome superb microvascular imaging (mSMI) in the ultrasonography and its magnified image. Blood flow is sharply captured by mSMI of ultrasonography at margin and inside of the mass (white arrow), which was not detected at all with conventional color or power Doppler. (c) Nonenhanced CT image. The mass shows constant density compared with other part of the kidney and thereby it is not clearly detected. The protrusion of the mass is not clearly observed due to its small size (white arrow). (d) Dynamic contrast‐enhanced CT image. A nodular lesion is clearly detected at the center of the right kidney (white arrow) [Color figure can be viewed at wileyonlinelibrary.com]

A 60‐year‐old male with ESRD due to T2DM had been receiving HD for 8 years. He was diagnosed as T2DM and hypertension when he was 30 years old. His renal function gradually declined and HD was introduced due to overhydration accompanied with nephrosis when he was 52 years old. He had been treated with weekly GLP‐1 receptor agonist (Dulaglutide) and long‐acting basal insulin (Insulin degludec). His glycoalbumin had been fluctuating around 20%–23%. Abdominal ultrasonography revealed oval mass at the center part of the right kidney (17 mm × 16 mm in diameter) and presented relatively low and homogeneous echo in the internal part (Figure 1(a)). Interestingly, mSMI of ultrasonography sharply captured blood flow at margin and inside of the mass, which was not detected at all with conventional color or power Doppler (Figure 1(b) and Video S1; its moving image). It was hard to detect the mass in nonenhanced CT as the mass showed constant density compared with other part of the kidney and the protrusion of the mass was not clear due to its small size (Figure 1(c)). Finally, abdominal dynamic contrast‐enhanced CT detected a nodular lesion at the center of the right kidney, which presented an early enhancement and washout pattern, leading to the diagnosis of an early stage RCC (Figure 1(d)).

## DISCUSSION

This is the first report to provide the possibility that mSMI is very promising for detecting an early stage RCC with high sensitivity in a HD patient with T2DM. If mSMI had not been used in the first screen by abdominal ultrasonography, dynamic contrast‐enhanced CT might not have been conducted, which must have led to the delay of diagnosis. It was previously reported that contrast‐enhanced ultrasonography using perflubutane (CEUS) was useful for the diagnosis of RCCs.^4^ On the other hand, mSMI of ultrasonography is completely noninvasive and could be used as an additional or alternative diagnostic technology to CT and/or CEUS for evaluating renal masses in dialysis patients as renal blood flow is often hard to detect. Furthermore, mSMI might be also useful when contrast enhancement is not obvious on CT and/or CEUS or contrast agents are contraindicated. Moreover, it has been reported that mSMI could effectively detect vascularity of renal malignant tumor than benign renal mass in nondialysis patients.^5^ However, as it is still unknown whether mSMI could effectively distinguish renal malignant tumor and benign renal mass in HD patients as well as nondialysis patients, accumulation of data will be needed to address this point. It has been reported that mSMI had the 63% sensitivity to detect indeterminate renal masses compared with other modalities such as color Doppler imaging or power Doppler imaging in nondialysis patients.^6^ In addition, it will need further investigation with a large number of subjects about the sensitivity and specificity of mSMI of ultrasonography for detection of early stage RCCs in HD patients.

In conclusion, we propose that mSMI of ultrasonography could become one of the very useful methods for detecting RCCs at an early stage with high sensitivity in HD patients with T2DM.

## CONFLICT OF INTEREST

There is no conflict of interest related to this manuscript.

## Supporting information

**Video S1**: Moving image of mSMI.

## References

[1] Graff RE, Sanchez A, Tobias DK, Rodriguez D, Barrisford GW, Blute ML, et al. Type 2 diabetes in relation to the risk of renal cell carcinoma among men and women in two large prospective cohort studies. Diabetes Care. 2018;41(7):1432–7. 29678810 10.2337/dc17-2518 PMC6014546

[2] Tsuzuki T, Iwata H, Murase Y, Takahara T, Ohashi A. Renal tumors in end‐stage renal disease: a comprehensive review. Int J Urol. 2018;25(9):780–6. 30066367 10.1111/iju.13759

[3] Xiao XY, Chen X, Guan XF, Wu H, Qin W, Luo BM. Superb microvascular imaging in diagnosis of breast lesions: a comparative study with contrast‐enhanced ultrasonographic microvascular imaging. Br J Radiol. 2016;89(1066):20160546. 27529640 10.1259/bjr.20160546 PMC5124819

[4] Hashimoto M, Ohkuma K, Akita H, Yamada Y, Nakatsuka S, Mizuno R, et al. Usefulness of contrast‐enhanced ultrasonography for diagnosis of renal cell carcinoma in dialysis patients: comparison with computed tomography. Medicine. 2019;98(47):e18053. 31764832 10.1097/MD.0000000000018053 PMC6882623

[5] Mao Y, Mu J, Zhao J, Zhao L, Xin X. The value of superb microvascular imaging in differentiating benign renal mass from malignant renal tumor: a retrospective study. Br J Radiol. 2018;91(1082):20170601. 29125337 10.1259/bjr.20170601 PMC5965799

[6] Leong JY, Wessner CE, Kramer MR, Forsberg F, Halpern EJ, Lyshchik A, et al. Superb microvascular imaging improves detection of vascularity in indeterminate renal masses. J Ultrasound Med. 2020;39(10):1947–55. 32309889 10.1002/jum.15299

